# Exploring the therapeutic potential of lipid-based nanoparticles in the management of oral squamous cell carcinoma

**DOI:** 10.37349/etat.2024.00272

**Published:** 2024-09-29

**Authors:** Anis Ahmad Chaudhary, Mohammad Fareed, Salah-Ud-Din Khan, Lina M. Alneghery, Mohammed Aslam, Arockia Alex, Md. Rizwanullah

**Affiliations:** IRCCS Istituto Romagnolo per lo Studio dei Tumori (IRST) “Dino Amadori”, Italy; ^1^Department of Biology, College of Science, Imam Mohammad Ibn Saud Islamic University (IMSIU), Riyadh 11623, Saudi Arabia; ^2^College of Medicine, AlMaarefa University, Diriyah, Riyadh 11597, Saudi Arabia; ^3^Department of Biochemistry, College of Medicine, Imam Mohammad Ibn Saud Islamic University (IMSIU), Riyadh 11623, Saudi Arabia; ^4^Pharmacy Department, Tishk International University, Erbil 44001, Kurdistan Region, Iraq; ^5^Molecular and Nanobiotechnology Laboratory (MNBL), Department of Biochemistry, Saveetha Medical College and Hospital, Saveetha Institute of Medical and Technical Sciences (SIMATS), Chennai 602105, Tamil Nadu, India; ^6^Drug Delivery and Nanomedicine Unit, Center for Global Health Research, Saveetha Medical College and Hospital, Saveetha Institute of Medical and Technical Sciences (SIMATS), Chennai 602105, Tamil Nadu, India

**Keywords:** Oral squamous cell carcinoma, pathogenesis, lipid-based nanoparticles, passive targeting, active targeting, cytotoxicity

## Abstract

Oral squamous cell carcinoma (OSCC) is a highly malignant and invasive tumor with significant mortality and morbidity. Current treatment modalities such as surgery, radiotherapy, and chemotherapy encounter significant limitations, such as poor targeting, systemic toxicity, and drug resistance. There is an urgent need for novel therapeutic strategies that offer targeted delivery, enhanced efficacy, and reduced side effects. The advent of lipid-based nanoparticles (LNPs) offers a promising tool for OSCC therapy, potentially overcoming the limitations of current therapeutic approaches. LNPs are composed of biodegradable and biocompatible lipids, which minimize the risk of toxicity and adverse effects. LNPs can encapsulate hydrophobic drugs, improving their solubility and stability in the biological environment, thereby enhancing their bioavailability. LNPs demonstrate significantly higher ability to encapsulate lipophilic drugs than other nanoparticle types. LNPs offer excellent storage stability, minimal drug leakage, and controlled drug release, making them highly effective nanoplatforms for the delivery of chemotherapeutic agents. Additionally, LNPs can be modified by complexing them with specific target ligands on their surface. This surface modification allows the active targeting of LNPs to the tumors in addition to the passive targeting mechanism. Furthermore, the PEGylation of LNPs improves their hydrophilicity and enhances their biological half-life by reducing clearance by the reticuloendothelial system. This review aims to discuss current treatment approaches and their limitations, as well as recent advancements in LNPs for better management of OSCC.

## Introduction

Oral squamous cell carcinoma (OSCC) is an invasive and highly malignant solid tumor associated with substantial mortality and morbidity rates. As a predominant subtype of head and neck carcinoma, OSCC ranks among the most prevalent malignant tumors globally, encompassing approximately 95% of all cases within this category [1, 2]. The pathogenesis of OSCC involves an ulceroproliferative lesion progressing through multiple stages before culminating in the manifestation of carcinoma. Notably, OSCC can affect various regions of the oral mucosa, spanning from the lips to the oropharynx. The primary etiological factors associated with OSCC include alcohol consumption, tobacco use, areca and betel chewing habits, human papillomavirus (HPV), and Epstein-Barr virus infections, dental issues such as sharp tooth crowns and residual roots, exposure to ultraviolet radiation, and genetic predisposition [3–5]. The majority of OSCC cases are diagnosed at advanced stages, significantly reducing survival rates. Current treatment modalities include surgery, radiotherapy, and chemoradiotherapy. However, the efficacy of these treatments varies depending on factors such as the stage of the disease, the patient’s overall health, and the tumor’s responsiveness to therapy. Conventional chemotherapy presents several major limitations, including poor targeting of anticancer drugs to cancer cells at the tumor site, resulting in low local drug concentration and reduced therapeutic efficacy. Moreover, issues such as drug resistance, systemic toxicity, and associated adverse effects further complicate treatment outcomes [6, 7]. Furthermore, most anticancer drugs are characterized by high lipophilicity, low aqueous solubility, limited dissolution in biological fluids, and low bioavailability which further limits their therapeutic efficacy [8, 9]. Therefore, exploring novel therapeutic strategies that offer targeted delivery, enhanced efficacy, and reduced toxicity is crucial for effective OSCC management.

Nanoparticles-based drug delivery systems offer an advanced and effective approach for the treatment of OSCC, providing several significant advantages over conventional therapies due to their unique properties and capabilities. These systems can encapsulate therapeutic agents, enhancing their solubility, stability, and bioavailability, which is crucial for most anticancer drugs that have poor water solubility and are prone to rapid degradation in the biological environment [10, 11]. The nanoscale size of these particles allows them to penetrate tumor tissues more effectively via the enhanced permeability and retention (EPR) effect. This effect exploits the leaky vasculature and poor lymphatic drainage characteristics of solid tumors, leading to higher drug concentrations at the target site thereby reducing the exposure of drug to healthy tissues, and reducing systemic toxicity. Nanoparticles have shown the potential to encapsulate more than one chemotherapeutic drug. The biocompatibility and biodegradability of nanoparticles further ensure safety and tolerability [12–14]. Among different classes of nanoparticles, lipid-based nanoparticles (LNPs) emerge as a promising platform with the potential to revolutionize OSCC treatment. LNPs are composed of lipids that are naturally occurring or biologically similar, making them biocompatible and biodegradable, which minimizes the risk of toxicity and adverse effects [15]. Many anticancer drugs have poor water solubility, but LNPs can encapsulate hydrophobic drugs, improving their solubility and stability in the biological environment, which enhances their bioavailability and therapeutic efficacy. LNPs demonstrate significantly higher ability to encapsulate lipophilic drugs than other nanoparticle types. LNPs offer excellent storage stability, minimal drug leakage, and controlled drug release, rendering them highly effective nanoplatforms for the delivery of different chemotherapeutic agents [16, 17]. Additionally, LNPs can be modified by complexing them with specific target ligands on their surface. This surface modification allows the active targeting of LNPs to the tumors in addition to the passive targeting mechanism [18]. Furthermore, the PEGylation of LNPs improves their hydrophilicity and extends their biological half-life by reducing clearance by the reticuloendothelial system (RES) [19, 20].

## Pathogenesis of OSCC

Unlike other cancers, OSCC typically presents as non-healing ulcers or lesions in the mouth and often spreads to cervical lymph nodes. Histologically, it is characterized by keratinizing squamous cells, sometimes forming keratin pearls. OSCC is mainly associated with tobacco use and alcohol consumption, which are the primary risk factors for these cancers [21, 22]. However, in recent years, the incidence of OSCC, particularly in the base of the tongue and tonsil subsites, has increased significantly, and this increase is associated with high-risk types of HPV infection [23]. This shift in the etiology of OSCC, from a tobacco-related cancer to an HPV-related cancer, has important implications for its epidemiology, clinical characteristics, and management. Moreover, HPV-related OSCC has emerged as a distinct subset of head and neck cancers, characterized by unique epidemiological, clinical, and molecular characteristics that differ from non-HPV-related cancers [24]. HPV-related OSCC is more common among middle-aged white males and is associated with sexual behavior as a risk factor, differing from conventional risk factors like tobacco and alcohol use [25]. Furthermore, HPV-related OSCC has been shown to have improved survival compared to HPV-unrelated OSCC, likely due to its distinct molecular and clinical characteristics [26].

The pathogenesis of OSCC involves a complex series of molecular events that begin with genetic and epigenetic alterations in oral epithelial cells, leading to uncontrolled cell proliferation, resistance to cell death, and invasive behavior. Key molecular changes include mutations in oncogenes and tumor suppressor genes. TP53 is one of the most frequently mutated genes in OSCC, resulting in the loss of its tumor-suppressive functions such as DNA repair, apoptosis, and cell cycle regulation [27]. The activation of oncogenes like cyclin D1 (CCND1) further drives cell cycle progression and proliferation [28]. Additionally, alterations in the NOTCH signaling pathway, which normally regulates cell differentiation, are commonly observed, contributing to abnormal cell growth and differentiation [29]. Epigenetic modifications, including DNA methylation and histone modifications, further dysregulate gene expression, silencing tumor suppressor genes and activating oncogenes [30].

Moreover, recent studies have highlighted the significant role of c-Myc in OSCC pathogenesis. c-Myc, a well-known oncogene, is frequently overexpressed in OSCC and plays a pivotal role in regulating cell survival, proliferation, and metabolism [31]. Marconi et al. [32] demonstrated that c-Myc overexpression in OSCC is associated with enhanced tumor progression through various molecular mechanisms. These mechanisms include the promotion of cell cycle progression, inhibition of apoptosis, and regulation of metabolic pathways, all of which contribute to the aggressive nature of OSCC. c-Myc also influences the tumor microenvironment by modulating the expression of genes involved in angiogenesis and immune evasion, further supporting tumor growth and metastasis.

Overexpression of growth factors like epidermal growth factor (EGF) and their receptors (e.g., EGFR) leads to enhanced signaling through pathways such as PI3K/AKT and RAS/RAF/MEK/ERK, promoting cell survival, proliferation, and angiogenesis [33, 34]. Inflammatory cytokines and chronic inflammation, often due to tobacco use, alcohol consumption, and HPV infection, create a microenvironment that supports tumor development by inducing reactive oxygen species (ROS) and DNA damage [35, 36]. Matrix metalloproteinases (MMPs) are upregulated, degrading the extracellular matrix and facilitating tumor invasion and metastasis [37]. Loss of cell adhesion molecules such as E-cadherin contributes to epithelial-mesenchymal transition (EMT), which endows cancer cells with increased migratory and invasive capabilities [38]. Moreover, alterations in the immune response, including the suppression of anti-tumor immunity and the presence of immunosuppressive cells such as regulatory T cells (Tregs), further enable tumor progression [39]. Angiogenesis, driven by factors like vascular endothelial growth factor (VEGF), is essential for providing nutrients and oxygen to the growing tumor [40]. Together, these molecular events create a highly adaptable and aggressive cancer phenotype, leading to the development and progression of OSCC.

## Current treatment approaches and their limitations

The current treatment approaches for OSCC include surgery, radiation therapy, chemotherapy, targeted therapy, and immunotherapy. Each method has specific applications, benefits, and limitations.

### Surgery

Surgery is often the primary treatment for OSCC. The primary goal of surgery is to completely excise the tumor along with a surrounding margin of healthy tissue to minimize the risk of local recurrence. This can involve procedures ranging from simple excisions to more complex operations like glossectomies (removal of part or all of the tongue) or mandibulectomies (removal of part of the jawbone). Reconstructive surgery often follows to restore function and appearance, using techniques like free flap reconstruction or grafting. However, surgery can significantly affect speech, swallowing, and facial appearance. It can lead to substantial morbidity and a reduced quality of life. There are also risks of local recurrence or metastasis, as well as complications such as infection and prolonged recovery [41, 42].

### Radiation therapy

Radiation therapy is a common adjuvant treatment for OSCC, used to destroy any remaining cancer cells after surgery or as a primary treatment in cases where surgery is not feasible. It can be delivered as external beam radiation therapy, which targets the tumor from outside the body, or brachytherapy, which involves placing radioactive sources close to or inside the tumor. Radiation therapy is particularly effective in reducing the risk of local recurrence and can also be used palliatively to relieve symptoms in advanced cases. Radiation therapy is associated with both acute and chronic side effects. Acute side effects include mucositis (inflammation of the mucous membranes), xerostomia (dry mouth), dysphagia, and skin reactions in the treated area. Chronic effects can include fibrosis (scarring) of tissues, osteoradionecrosis (bone damage), and an increased risk of secondary cancers [43, 44].

### Chemotherapy

Chemotherapy involves the use of cytotoxic drugs to kill rapidly dividing cancer cells. In OSCC, it is typically used in combination with radiation therapy (chemoradiation) to enhance the effectiveness of treatment or as palliative care in advanced cases where curative treatment is no longer possible. The use of chemotherapy is limited by its significant toxicity profile. Patients often experience side effects such as nausea, vomiting, bone marrow suppression (leading to an increased risk of infection and anemia), nephrotoxicity (kidney damage), and neurotoxicity (nerve damage). The effectiveness of chemotherapy can be compromised by the development of drug resistance within the tumor, reducing its impact on overall survival [45–47].

### Targeted therapy

Targeted therapy is a more personalized approach to cancer treatment, involving drugs designed to specifically target molecular pathways involved in tumor growth and progression. In OSCC, one of the most common targets is the EGF receptor (EGFR), which is overexpressed in many cases. Cetuximab, an EGFR inhibitor, is often used in combination with radiation therapy or chemotherapy to block these signals and inhibit tumor growth. However, the effectiveness of targeted therapy is contingent on the presence of specific molecular markers within the tumor, meaning that not all patients will benefit from this approach. Additionally, tumors can develop resistance to targeted therapies, either through mutations in the target or activation of alternative signaling pathways. Another significant limitation is the high cost of targeted therapies, which can limit accessibility, particularly in resource-limited settings [48, 49].

### Immunotherapy

Immunotherapy represents a newer approach to treating OSCC by harnessing the patient’s immune system to recognize and destroy cancer cells. Immune checkpoint inhibitors, such as pembrolizumab and nivolumab, are designed to block proteins like PD-1/PD-L1 that tumors use to evade the immune system. By inhibiting these checkpoints, immunotherapy can enhance the immune response against cancer cells. However, only a subset of patients respond to immunotherapy, and the reasons for this variability are not fully understood. Immunotherapy can cause significant immune-related adverse effects, such as inflammation of organs. Its effectiveness often depends on the presence of certain biomarkers, like PD-L1 expression, limiting its use to patients who test positive for these markers [50, 51].

## Lipid-based nanoparticles (LNPs) for the treatment of OSCC

**Figure 1:**
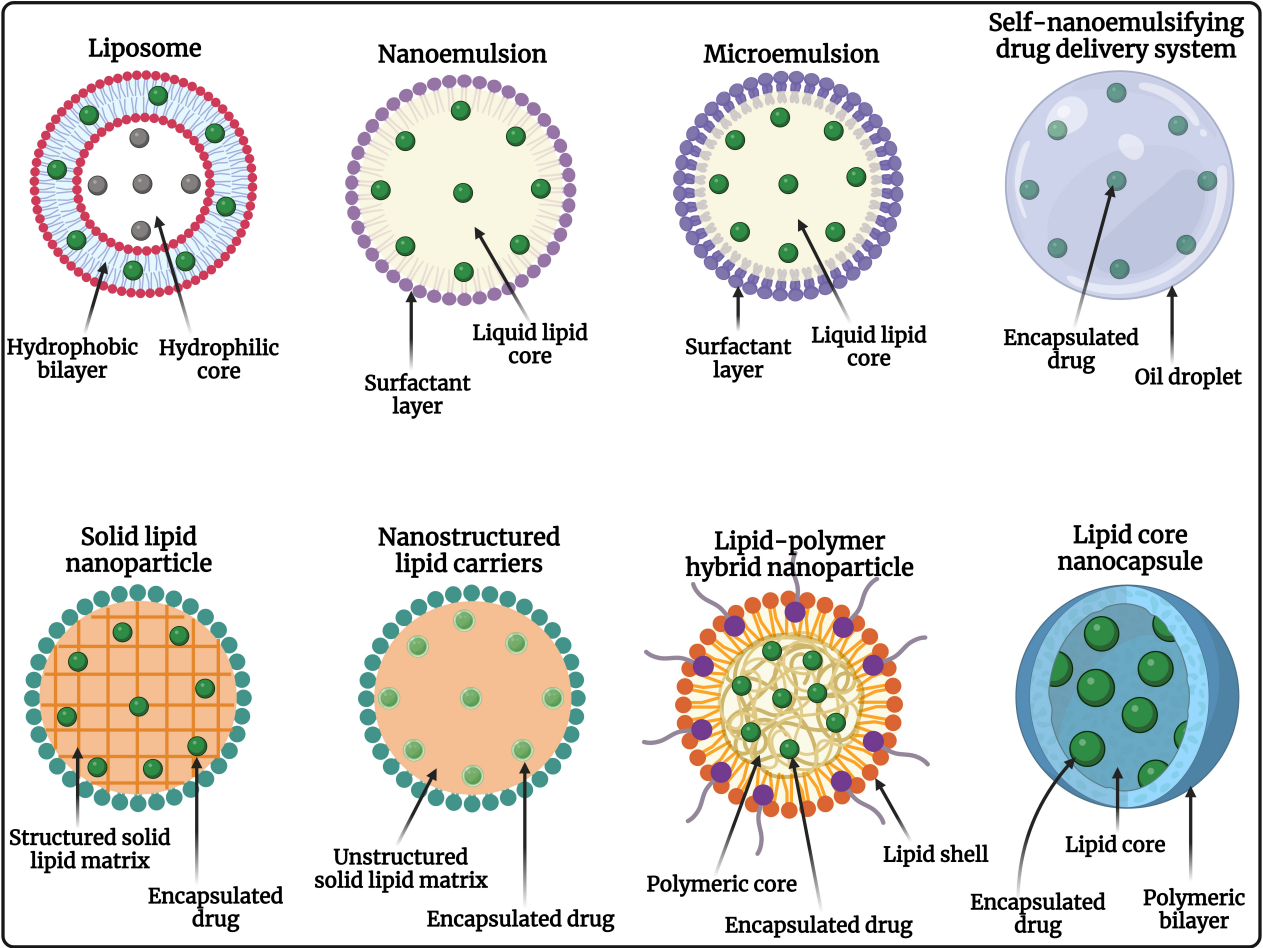
Diagrammatic representation of different types of LNPs used for chemotherapeutic drug delivery against OSCC. OSCC: oral squamous cell carcinoma. Created in BioRender. Rizwanullah, M. (2024) BioRender.com/n58m919

**Figure 2:**
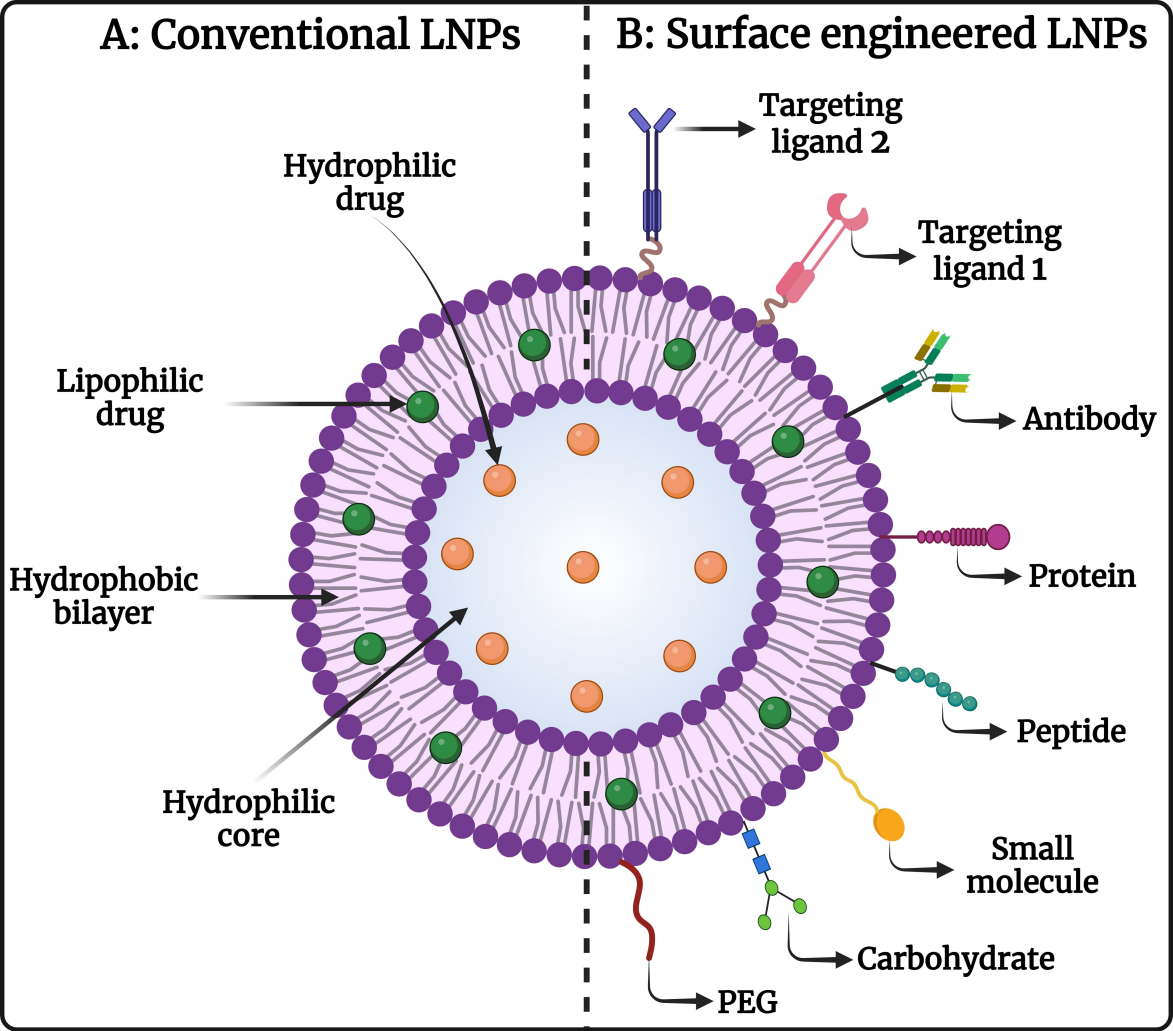
Image representing (A) conventional LNPs and (B) surface-engineered LNPs for chemotherapeutic drug delivery against OSCC. OSCC: oral squamous cell carcinoma. Created in BioRender. Rizwanullah, M. (2024) BioRender.com/g76a635

LNPs are composed of lipids that are naturally occurring or biologically similar, making them biocompatible and biodegradable, which minimizes the risk of toxicity and adverse effects. Most anticancer drugs have poor water solubility, but LNPs can encapsulate hydrophobic drugs, improving their solubility and stability in the biological environment, which enhances their bioavailability and therapeutic efficacy. Different types of LNPs are diagrammatically represented in Figure 1**.** LNPs can be engineered to release their payloads in a controlled manner, ensuring a sustained therapeutic effect, reducing the frequency of dosing, and improving patient compliance. Encapsulation of drugs in LNPs can enhance their bioavailability by protecting them from degradation and clearance by the immune system, resulting in higher concentrations of the drug reaching the tumor site [52–54]. Surface functionalization with ligands such as antibodies, peptides, or small molecules enables LNPs to specifically target cancer cells, enhancing the accumulation of the drug at the tumor site, and reducing off-target effects. Coating the surface of LNPs with polyethylene glycol (PEG) or other hydrophilic polymers provides a “stealth” characteristic, allowing them to evade the RES and prolong their circulation time in the bloodstream [55, 56]. LNPs exploit the EPR effect of tumors, allowing them to preferentially accumulate in tumor tissues due to leaky vasculature and poor lymphatic drainage. By delivering drugs specifically to the tumor site, LNPs minimize the exposure of healthy tissues to toxic drugs, reducing systemic side effects and improving the therapeutic index [57]. LNPs can co-deliver multiple therapeutic agents, including chemotherapeutics and siRNA, to overcome drug resistance mechanisms in cancer cells, enhancing anticancer efficacy by synergistic activity. Further, it allows targeted therapies, and immunomodulators, providing a multi-faceted approach that increases the likelihood of treatment success. The diagrammatic representation of conventional LNPs and surface-engineered LNPs is depicted in Figure 2. The ability to deliver drugs orally or via less invasive routes improves patient comfort and compliance compared to traditional intravenous chemotherapy [58, 59]. Therefore, LNPs represent a versatile and effective strategy for the treatment of OSCC, improving therapeutic outcomes, reducing side effects, and offering a platform for advanced combination therapies in the management of OSCC.

## Targeting approaches of LNPs

**Figure 3:**
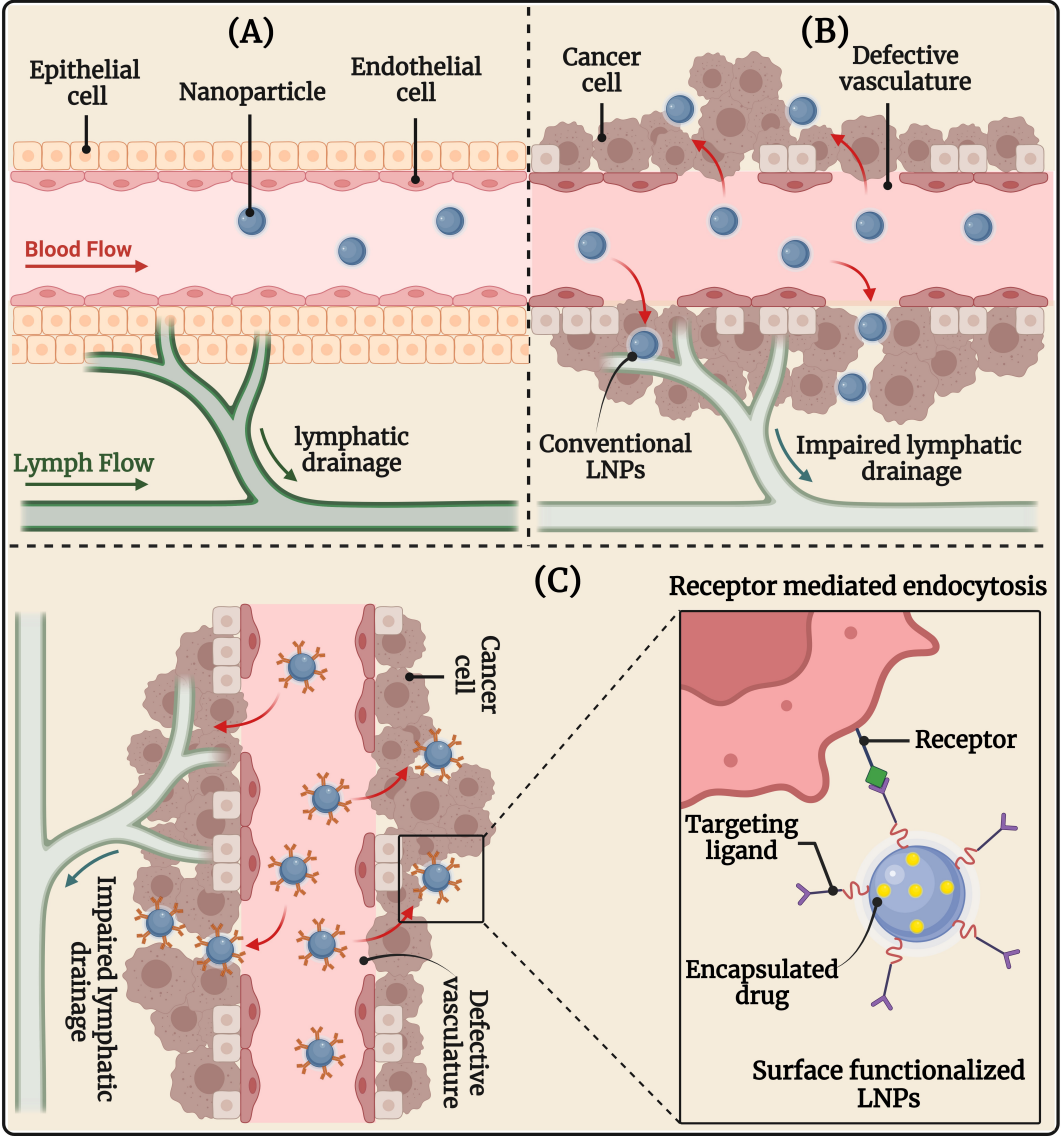
The image illustrates (A) healthy tissue and vasculature; (B) passive targeting of conventional LNPs, and (C) active targeting of surface-engineered LNPs in OSCC therapy. OSCC: oral squamous cell carcinoma. Created in BioRender. Rizwanullah, M. (2024) BioRender.com/c06n678s

Targeted drug delivery of LNPs represents several major advantages including (i) protection of healthy cells from the cytotoxic compounds, (ii) improved accumulation of the drug in the tumor thereby reducing dose-related cytotoxicity to normal healthy cells, and (iii) improved bioavailability and therapeutic efficacy. The LNPs can either be targeted via passive or active mechanisms as demonstrated in Figure 3.

### Passive targeting approach

Nanoparticles accumulate in tumors primarily due to the EPR effect. The EPR effect is a phenomenon where certain sizes of molecules, like nanoparticles, tend to accumulate more in tumor tissue than in normal tissues. This effect is mainly due to the unique characteristics of the tumor vasculature. When a solid tumor grows beyond a certain size, the existing normal blood vessels can no longer supply the necessary oxygen and nutrients for continued growth. This hypoxic environment triggers the release of growth factors that stimulate angiogenesis, leading to the formation of new blood vessels from the surrounding capillaries [60, 61]. The angiogenic process in tumors results in the development of new blood vessels that are often irregular, with a discontinuous endothelium and missing basal membrane. These newly formed tumor blood vessels have gaps or fenestrations that can range from 0.2 µm to 2 µm, depending on the tumor type, environment, and location. These fenestrations in the abnormal blood vessels allow blood components, including nanoparticles, to easily pass into the tumor interstitium and be retained for a longer period. This phenomenon, known as the EPR effect, is a key factor in the passive targeting of nanoparticles to tumors [62, 63].

The EPR effect facilitates the preferential accumulation of nanoparticles in tumor tissues due to their size, which is smaller than the gaps between the endothelial cells of tumor blood vessels. This size allows them to penetrate and become entrapped in the tumor tissue, leading to enhanced retention. This phenomenon forms the basis of the passive targeting mechanism of nanoparticles [64]. Despite its potential, the EPR effect is not universally effective across all tumor types. Factors such as tumor heterogeneity, variability in the extent of angiogenesis, and differences in the size and density of fenestrations can affect the efficiency of nanoparticle delivery. Additionally, the presence of enhanced interstitial fluid pressure in tumors can further hinder the penetration and distribution of nanoparticles [65, 66]. Therefore, while the EPR effect provides a promising mechanism for targeted drug delivery, it also presents challenges that must be addressed to optimize the efficacy of nanoparticle-based therapies.

### Active targeting approach

Active targeting, also called ligand-mediated targeting, involves the interaction of ligand-decorated nanoparticles with specific ligands, such as antibodies, peptides, or small molecules, that can bind to receptors overexpressed on the surface of cancer cells [67, 68]. This binding enhances the precision of drug delivery, ensuring that the therapeutic agents are more selectively delivered to the tumor cells via receptor-medicated endocytosis while minimizing exposure to healthy tissues. Most cancer cells overexpress specific types of receptors, which can serve as sites for the active targeting of ligand-functionalized nanoparticles [69, 70]. This approach reduces off-target effects and systemic toxicity, potentially improving therapeutic outcomes. Additionally, this strategy offers an alternative approach to addressing multi-drug resistance (MDR) since proteins that induce resistance, such as P-glycoprotein (P-gp), are unable to expel nanoparticle-bound drugs that have been internalized through the endocytic pathway [71, 72].

## Different LNPs for the treatment of OSCC

### Liposomes

Liposomes were initially studied by Bangham et al. [73] in 1961, with the first article on liposomes was published in 1965. Since then, liposomes have become prevalent delivery systems for a wide range of anticancer agents [74]. Liposomes consist of bilayer structures that exhibit physiological compatibility akin to human cell membranes, enhancing their compatibility with cellular membranes. The diameter of liposomes, which varies based on their lipid composition and preparation techniques, ranges from approximately 20 nm to over 1 μm [75]. Liposomes offer multiple advantages, such as enhancing the pharmacokinetic profiles of encapsulated drugs and extending their circulation time. Due to the EPR effect, they enable passive targeting and accumulation in tumor and inflamed tissues. Liposomes also mitigate systemic toxicity linked to free drugs, improve drug solubility, and facilitate the slow and sustained release of the encapsulated therapeutic agents [76, 77]. In this context, El-Hamid et al. [78] investigated the therapeutic potential of doxorubicin (DOX)-encapsulated liposomes (Doxil) against OSCC. Doxil represents a significantly higher apoptosis of CAL-27 cells than free DOX. The Doxil represents 2.72 times higher caspase-3 levels compared to the free DOX. Further, Doxil treatment revealed much higher (41%) c-Myc mRNA inhibition in comparison with the free DOX (27%) in the treatment of CAL-27 cells. In another study, Hariharan et al. [79] developed erlotinib-encapsulated chitosan-coated liposome-based gels for improved therapeutic efficacy against OSCC after local administration. The developed liposomal gel represents the vesicle size (VS), polydispersity index (PDI), entrapment efficiency (EE), and zeta potential (ZP) of ~ 200 nm, 0.38, > 70%, and > +27 mV respectively. The developed liposomal gel represents the controlled release of the encapsulated drug (erlotinib) for up to 36 h and revealed excellent stability. MTT assay results revealed that the developed liposomal gels represent ~ 2 times reduction in IC_50_ value against KB 3-1 cells in comparison with the free DOX. Further, the developed liposomal gel showed much better in vivo therapeutic efficacy in OSCC-bearing Sprague Dawley (SD) rats compared to the free drug after local application.

### Nanoemulsion

Nanoemulsions are emulsions with nanoscale dimensions, either water-in-oil or oil-in-water, which can be utilized for drug delivery to tumor sites [80]. Their hydrophobic core allows them to encapsulate poorly water-soluble drugs effectively. Moreover, nanoemulsions are composed of safe excipients, enhancing their stability and safety as drug delivery systems [81]. They offer several benefits, including biocompatibility, non-immunogenicity, biodegradability, small size, large surface area, ease of fabrication, sustained and controlled release, and thermodynamic stability [82]. In this context, Tamane et al. [83] developed berberine and 5-fluorouracil (5-FU) co-encapsulated nanoemulsion-based mucoadhesive buccal gels for the effective management of OSCC. The developed combinatorial nanoemulsion represents the droplet size (DS), PDI, ZP, and EE of 37.7 nm, 0.126, ‒0.7 mV, and 94.67% respectively. The developed nanoemulsion-based gels represent excellent mucoadhesive strength and better ex vivo permeation across buccal porcine mucosa. The developed nanoemulsion-based gels revealed 5-fold improved apoptotic activity against the OC cell line compared to the free 5-FU. Further, the developed gels represent synergistic anticancer activity against AW13516 oral cancer cells. In addition, the developed gel showed excellent biocompatibility in New Zealand rabbits even after 7 days of buccal administration. In another study, Srivastava et al. [84] developed 5-FU, and curcumin (CUR) encapsulated nanoemulsion for improved therapeutic efficacy against OSCC. The developed nanoemulsion represents the DS, PDI, and ZP of 196 ± 6.35 nm, 0.29, and ‒22.50 ± 4.87 mV, respectively. The developed nanoemulsion showed excellent stability and sustained release of drugs for up to 96 h. The combinatorial nanoemulsion represents a much higher synergistic anticancer activity against both SCC090 and SCC152 oral cancer cells compared to the single drug-loaded nanoemulsion.

### Microemulsions

Microemulsions are colloidal dispersions consisting of oil, surfactants, and water, structurally resembling nanoemulsions. However, their production is more cost-effective and scalable. They differ from nanoemulsions in DS, thermodynamic stability, surfactant-to-oil ratio, and the amount of external energy needed for preparation. Microemulsions are thermodynamically stable, necessitating a higher surfactant-to-oil ratio and minimal external energy [85, 86]. Encapsulation of different anticancer molecules within the microemulsions has demonstrated promising outcomes in cancer therapy [87]. In a study, Lin et al. [88] developed a CUR-encapsulated microemulsion to achieve better therapeutic efficacy against OSCC. The developed microemulsion revealed the DS and ZP of 41 ± 3 nm and −42.12 ± 1.26 mV, respectively. The formulated microemulsion demonstrated remarkable stability and exhibited a controlled drug release profile. Further, drug-loaded microemulsion represented much higher anticancer activity against OSCC-25 oral cancer cells than pure drugs. Thus, the fabrication of drug-encapsulated microemulsion presents a promising strategy for the treatment of OSCC.

### Self-nano emulsifying drug delivery system (SNEDDS)

SNEDDS are anhydrous, homogenous liquid mixtures composed of oil, surfactant, drug, and a co-emulsifier or solubilizer. Upon dilution with water and gentle stirring, these mixtures spontaneously form oil-in-water nanoemulsions with DS typically around 200 nm or smaller. The spontaneous emulsification process happens when the entropy change that promotes dispersion exceeds the energy required to increase the surface area of the emulsion [89, 90]. SNEDDS can enhance the solubility, bioavailability, and stability of poorly water-soluble drugs. The development of SNEDDS for cancer chemotherapy holds promise for overcoming the limitations associated with conventional drug delivery systems and improving the efficacy and safety of anticancer therapies [91, 92]. In this context, Rizg et al. [93] developed lovastatin (LV) encapsulated eucalyptus oil-based SNEDDS (LV-SNEDDS) for the treatment of tongue carcinoma. The developed LV-SNEDDS revealed the DS, PDI, and ZP of 85 ± 2 nm, 0.371 ± 0.006, and 21.6 ± 2.1 mV, respectively. The developed LV-SNEDDS exhibited biphasic drug release profiles with adequate stability. The LV-SNEDDS revealed much higher cytotoxicity against HSC3 cells than the free drug.

### Solid lipid nanoparticles (SLNs)

SLNs consist of a solid lipid core, mainly containing glycerides, fatty acids, sterols, or waxy molecules that are stabilized by emulsifiers [94]. SLNs present notable advantages including low toxicity, enhanced drug bioavailability, and the potential to encapsulate both hydrophilic and lipophilic drugs, facilitating large-scale production [95]. The quality of SLN preparations heavily depends on their molecular structure, which is influenced by both composition and preparation methods [96]. Moreover, SLNs can surmount various physiological barriers impeding drug delivery to tumors [97]. In terms of cell delivery, SLNs enhance drug targeting through passive mechanisms leveraging the tumor microenvironment, active mechanisms via surface modifications, and co-delivery strategies [98]. In this context, Arana et al. [99] developed all-trans-retinoic acid (ATRA) encapsulated phosphatidylethanolamine-PEG (PE-PEG) functionalized SLNs (ATRA-PE-PEG-SLNs) to achieve better therapeutic efficacy against OSCC. The developed ATRA-PE-PEG-SLNs represented the particle size (PS), PDI, and ZP of < 150 nm, < 0.4, and > ‒35 mV, respectively. Surface modification of SLNs with PE-PEG significantly improves the cellular uptake of nanoparticles in SCC-25 cells which was further confirmed by confocal microscopy. Further, the developed nanoparticles revealed a much higher dose and time-dependent cytotoxicity against SCC-25 cells than the free drug. In another study, Bharadwaj and Medhi [100] fabricated paclitaxel (PTX) encapsulated folic acid (FA) functionalized SLNs (PTX-FA-SLNs) for targeted treatment of OSCC. The developed PTX-FA-SLNs revealed the PS, PDI, ZP, and EE of 99.12 ± 2.1 to 603.76 ± 1.9 nm, 0.31 to 0.51, ‒19.9 ± 1.2 to ‒27.2 ± 1.1 mV, and 56.4% to 69.3%, respectively. The developed PTX-FA-SLNs exhibited 2.21 times improved bioavailability after intravenous administration in Swiss Albino mice in comparison with the free PTX. Further, the PTX-FA-SLNs represent much better in vivo therapeutic efficacy against 4-nitoquinoline-1-oxide (4-NQO) induced oral cancer-bearing Swiss Albino mice in comparison with the free PTX.

### Nanostructured lipid carriers (NLCs)

NLCs are also known as the second-generation SLNs. NLCs are prepared with a lipidic-liquid interface, usually consisting of oils, and typically embedded within a solid core such as an SLN [101]. NLCs can further enhance the solubility and stability of certain compounds that are not well solubilized in the solid core of SLNs, making them a suitable delivery vector for the potential co-encapsulation of drugs in either the oil phase, the solid phase, or both depending on the matrix mixture and drug properties [102, 103]. NLCs represent almost all the advantages of SLNs and these properties make them ideal candidates for anticancer drug delivery [104]. In this context, Shete et al. [105] developed silymarin-encapsulated NLC-based mucoadhesive gels for the localized treatment of OSCC. The NLCs-based gel revealed the PS, PDI, ZP, and EE of 315.5 ± 0.10 nm, 0.341 ± 0.01, 14.01 ± 0.21 mV, and 71.05 ± 0.05%, respectively. The developed NLCs-based gel revealed excellent stability for 90 days and showed a controlled drug release profile for 5 days. The NLCs-based mucoadhesive gel represents excellent mucoadhesive properties and permeation across the mucosal membrane. Further, the developed gel exhibited much higher cytotoxicity against KB cells than the free drug. In another study, Chaudhari et al. [106] developed quercetin and piperine co-encapsulated NLCs to achieve synergistic therapeutic efficacy against OSCC. The developed NLCs revealed the PS, PDI, ZP, and EE of 128.10 ± 32.02 nm, 0.16 ± 0.050, ‒17.06 ± 4.73 mV, and > 85%, respectively. The developed NLCs showed very fast cellular internalization in FaDu cells with a ~ 90% localization rate. The MTT assay result suggested a much better cytotoxic potential by showing synergistic activity of the developed formulation against FaDu cells in comparison with the individual drug or combination of drugs. The NLCs represent higher depolarisation of the mitochondrial membrane with higher induction of apoptosis which was further confirmed by flow cytometry.

### Lipid polymer hybrid nanoparticles (LPHNPs)

LPHNPs represent a progressive evolution in nanoparticle technology, leveraging the advantageous features of both polymeric nanoparticles and liposomes [107]. These PLHNPs integrate biocompatible polymers and biomimetic lipids, endowing them with remarkable versatility in delivering chemotherapeutic agents with diverse physicochemical properties. The hybrid architecture of PLHNPs offers numerous benefits, including small particle size, the capability to encapsulate multiple anticancer drugs, high drug loading capacity, and tailored drug release profiles [108, 109]. Additionally, surface modification of PLHNPs enhances the therapeutic efficacy of chemotherapeutic agents through selective targeting of tumor tissue while mitigating side effects by reducing nonspecific biodistribution [110, 111]. In this context, Wu et al. [112] developed DOX and triptolide co-encapsulated redox-sensitive LPHNPs synergistic OSCC treatment. The developed LPHNPs represent the PS, PDI, ZP, and EE of 120 ± 5 nm, 0.12 ± 0.04, ‒9.7 ± 0.8 mV, and ~ 60%, respectively. The developed LPHNPs exhibited synergistic cytotoxicity against KB cells. Moreover, the developed LPHNPs revealed significantly higher therapeutic efficacy in H22-tumor bearing BALB/c-nu nude mice xenograft in comparison with single drug-loaded LPHNPs and free individual drugs. In another study, the same researchers developed DOX-encapsulated redox-sensitive LPHNPS to treat OSCC [113]. The PS, PDI, ZP, and EE of the developed LPHNPs were observed to be 100–120 nm, 0.12, −8.5 ± 2.4 mV, and 82.5 ± 2%, respectively. Findings obtained from flow cytometry, confocal imaging, and in vitro cytotoxicity assessments demonstrated that targeted LPNPs substantially augmented cellular uptake and elicited greater cytotoxic effects against KB cells compared to both non-targeted redox-sensitive and targeted redox-insensitive controls. Furthermore, in vivo studies revealed a higher accumulation of drugs in the tumor and remarkably reduced tumor growth in comparison with the non-targeted redox-sensitive and targeted redox-insensitive controls.

### Lipid-core nanocapsules (LNCs)

LNCs are categorized within the vesicular colloidal nanocarrier class, comprising polymeric bilayers enveloping lipid cores containing a blend of solid and liquid lipids [114]. LNCs have garnered significant interest as potential carriers for hydrophobic drugs across various applications [115, 116]. Incorporating both liquid and solid lipids in LNC development offers distinct advantages over alternative colloidal vesicular nanocarriers, including enhanced stability, controlled drug release kinetics, and the potential for site-specific targeting [117, 118]. In this context, Ortega et al. [119] developed CUR-encapsulated chitosan-coated LNCs for buccal administration for the treatment of OSCC. The developed mucoadhesive nanocapsules represent the PS, PDI, ZP, and EE of 179 ± 48 nm, 0.26 ± 0.01, +19.0 ± 3.18 mV, and 99.88%, respectively. The chitosan-coated lipid nanocapsules exhibited higher mucoadhesion and drug retention on porcine buccal mucosa compared to the uncoated lipid nanocapsules. The mucoadhesive lipid nanocapsules were observed to be non-irritant and showed controlled drug release profiles for up to 48 h. The developed mucoadhesive lipid nanocapsules exhibited significantly higher dose and time-dependent cytotoxicity against SCC-25 cells compared to the free drug. From the above discussion and extensive review of literature, as summarized in Table 1, it can be inferred that the encapsulation of chemotherapeutic drug delivery using different LNPs can be a great nanoplatform for the effective management of OSCC.

**Table 1:** Different chemotherapeutic drug-loaded LNPs for the effective management of OSCC

**Nanoparticles**	**Drug encapsulated**	**Pharmaceutical attributes**	**Cell line/animal model**	**Major outcomes**	**Reference**
Liposomes	Doxorubicin	PS: NA PDI: NA ZP: NA EE: NA	CAL-27 cells	Significantly higher apoptosis of CAL-27 cells by showing 2.72 times higher caspase-3 levels compared to the free drug. Much higher c-Myc mRNA inhibition in comparison with the free DOX in the treatment of CAL-27 cells.	[78]
Liposomes	Erlotinib	PS: ~ 200 nm PDI: 0.38 ZP: +27 mV EE: > 70%	KB 3-1 cells/Sprague Dawley (SD) rats	Excellent stability and controlled release for u to 36 h. ~ 2 times reduction in IC_50_ value against KB 3-1 cells. Much better therapeutic efficacy against KB 3-1 tumor-bearing SD rats.	[79]
Nanoemulsion	Berberine & 5-fluorouracil	PS: 37.7 nm PDI: 0.126 ZP: ‒0.7 mV EE: 94.67%	OC cells & AW13516 cells	Excellent mucoadhesive strength and higher ex vivo permeation of drugs across buccal porcine mucosa. 5-Fold improved apoptotic activity against the OC cell line compared to the free 5-fluorouracil. Synergistic anticancer activity against AW13516 oral cancer cells.	[83]
Nanoemulsion	Curcumin & 5-fluorouracil	PS: 196 ± 6.35 nm PDI: 0.29 ZP: ‒22.50 ± 4.87 mV EE: NA	SCC090 & SCC152 cells	Excellent stability and controlled release of drug for up to 96 h. Represented synergistic anticancer activity against both cell lines.	[84]
Microemulsion	Curcumin	PS: 41 ± 3 nm PDI: NA ZP: −42.12 ± 1.26 mV EE: NA	OSCC-25 cells	Much higher anticancer activity against OSCC-25 oral cancer cells compared to the free drug. The addition of ultrasound significantly improved the therapeutic effects.	[88]
Self-nanoemulsifying drug delivery system	Lovastatin	PS: 85 ± 2 nm DPI: 0.371 ± 0.006 ZP: +21.6 ± 2.1 mV EE: NA	HSC3 cells	Biphasic drug release profiles. Significantly higher cytotoxicity in comparison to the free drug.	[93]
Solid lipid nanoparticles	All-trans-retinoic acid	PS: < 150 nm PDI: < 0.4 ZP: > ‒35 mV EE: NA	SCC-25 cells	Improved cellular uptake. Higher dose and time-dependent cytotoxicity compared to the free drug.	[99]
Solid lipid nanoparticles	Paclitaxel	PS: 99.12 nm to 603.76 nm PDI: 0.31 to 0.51 ZP: ‒19.9 mV to ‒27.2 mV EE: 56.4% to 69.3%	Swiss Albino mice	2.21 times improved bioavailability after intravenous administration. Much higher therapeutic efficacy against 4-nitoquinoline-1-oxide (4-NQO) induced oral cancer-bearing Swiss Albino mice.	[100]
Nanostructured lipid carriers	Silymarin	PS: 315.5 ± 0.10 nm PDI: 0.341 ± 0.01 ZP: +14.01 ± 0.21 mV EE: 71.05 ± 0.05%	KB cells	Represents excellent stability and controlled drug release for up to 5 days. Strong mucoadhesive properties and higher permeation across the mucosal membrane. Significantly higher cytotoxicity.	[105]
Nanostructured lipid carriers	Quercetin & Piperine	PS: 128.10 ± 32.02 nm PDI: 0.16 ± 0.050 ZP: ‒17.06 ± 4.73 mV EE: > 85%	FaDu cells	Very fast cellular internalization and localization. Represents synergistic cytotoxicity against FaDu cells. Represent higher depolarisation of the mitochondrial membrane with higher induction of apoptosis.	[106]
Lipid polymer hybrid nanoparticles	Doxorubicin & Triptolide	PS: 120 ± 5 nm PDI: 0.12 ± 0.04 ZP: ‒9.7 ± 0.8 mV EE: ~ 60%	KB cells/H22-tumor-bearing BALB/c-nu mice	Drug release at tumoric pH. Higher synergistic cytotoxicity against KB cells. Much higher in vivo anti-tumor efficacy.	[112]
Lipid polymer hybrid nanoparticles	Doxorubicin	PS: 100–120 nm PDI: 0.12 ZP: −8.5 ± 2.4 mV EE: 82.5 ± 2%	KB cells/H22-tumor-bearing BALB/c-nu mice	pH-dependent drug release. Higher cytotoxicity against KB cells compared to the free drug. Much better in vivo therapeutic efficacy.	[113]
Lipid-core nanocapsules	Curcumin	PS: 179 ± 48 nm PDI: 0.26 ± 0.01 ZP: +19.0 ± 3.18 mV EE: 99.88%	SCC-25 cells	Higher mucoadhesion and drug retention on porcine buccal mucosa. The formulation was non-irritant and represents controlled drug release profiles for up to 48 h. Significantly higher dose and time-dependent cytotoxicity against than the free drug.	[119]

PS: particles size; PDI: polydispersity index; ZP: zeta potential; EE: entrapment efficiency; OSCC: oral squamous cell carcinoma; LNPs: lipid-based nanoparticles

## LNPs for photothermal therapy of OSCC

LNPs have emerged as promising candidates for photothermal therapy (PTT) due to their biocompatibility, versatility in encapsulating various payloads, and ability to preferentially accumulate in tumor tissues [120]. LNPs such as liposomes, can be engineered to incorporate near-infrared absorbing materials, such as gold or carbon-based nanomaterials, that can efficiently convert light energy into heat [121, 122]. When these nanoparticles are selectively delivered to the tumor site and exposed to near-infrared light, the localized heating can induce apoptosis and necrosis of cancer cells while sparing healthy surrounding tissues [123]. Nanoparticle-mediated PTT works by leveraging the EPR effect, where nanoparticles extravasate and accumulate in the tumor microenvironment. Once the nanoparticles have localized to the tumor site, near-infrared light is applied, which is absorbed by the nanoparticles and converted into heat, leading to the selective destruction of cancer cells. This approach is advantageous as it can be targeted to the tumor site, minimizing off-target effects and damage to healthy tissues [124]. In this context, Xu et al. [125] developed a thermally responsive liposome (PITRL) by encapsulating the water-soluble immunostimulatory molecule polyinosinic acid within the hydrophilic core of liposomes and incorporating the organic dye ICG into the lipid bilayer. Upon exposure to NIR irradiation at 808 nm, ICG converts light energy into heat, generating a photothermal effect. This temperature increase in PITRL triggers the release of polyinosinic acid, which subsequently activates dendritic cells in tumor-draining lymph nodes. The activation of these dendritic cells plays a crucial role in inhibiting the growth of lung metastasis following the venous transplantation of cancer cells [125].

Moreover, the LNPs can be further functionalized to achieve targeted delivery and enhanced tumor accumulation. For instance, the incorporation of ligands or antibodies that recognize specific receptors overexpressed on oral cancer cells can facilitate active targeting and improve the therapeutic efficacy of the PTT [126]. In addition to the photothermal effect, the LNPs can also be used to co-deliver chemotherapeutic agents, thereby enabling a multimodal approach that combines hyperthermia and chemotherapy. The synergistic effects of these combined modalities have been shown to enhance the overall anti-tumor activity and overcome the limitations of individual therapies [127].

## LNPs for photodynamic therapy of OSCC

Photodynamic therapy (PDT) has emerged as a promising treatment modality for various types of cancer, including OSCC. A critical aspect of effective PDT is the careful selection and design of photosensitizer molecules that generate cytotoxic ROS upon light activation, leading to the targeted destruction of cancer cells [128]. LNPs have shown strong potential as a delivery platform for photosensitizers, providing enhanced solubility, higher tumor targeting, and improved light penetration, all of which are essential for maximizing the therapeutic outcomes of PDT [129].

Common photosensitizers used in PDT, such as porphyrins, phthalocyanines, and chlorins, have been extensively studied for their effectiveness in treating various cancers. These compounds are well-known for their ability to produce singlet oxygen-a highly reactive form of oxygen that induces cell death when exposed to specific wavelengths of light. However, these photosensitizers often suffer from limitations such as poor water solubility, low bioavailability, and non-specific distribution within the body [130, 131].

Encapsulating these photosensitizers within LNPs can overcome many of these challenges. LNPs can enhance the solubility of photosensitizers, improve their stability in biological environments, and enable controlled release at the tumor site. Moreover, LNPs can be engineered to target tumor tissues specifically, reducing off-target effects and minimizing damage to healthy cells [132, 133]. When common photosensitizers like porphyrins or chlorins are combined with LNPs-based delivery systems, the resulting nano-complex offers a more effective and targeted approach to PDT. This combination addresses conventional PDT’s limitations such as poor water solubility, long-term phototoxicity, and low tumor targeting efficiency, and enhances the overall therapeutic efficacy. As a result, the development of photosensitizers encapsulated LNPs represents a significant advancement in the treatment of OSCC [134, 135].

## Challenges ahead and future outlook

LNPs have emerged as a promising modality for OSCC therapy, offering several advantages over conventional treatment approaches. The unique properties of LNPs, including their biocompatibility, biodegradability, and ability to encapsulate and deliver hydrophobic drugs, made them a potent tool in the fight against OSCC. LNPs can overcome the limitations associated with traditional chemotherapy, such as poor drug solubility, limited bioavailability, and systemic toxicity. Additionally, the ability of LNPs to penetrate tumor tissues through the EPR effect allows for higher drug concentrations at the tumor site. One of the most exciting aspects of LNPs is their potential for targeted drug delivery. Through surface modifications, LNPs can be engineered to actively target specific tumor cells, thereby enhancing the specificity and effectiveness of the treatment. This targeted approach increases the therapeutic index of anticancer drugs and reduces the adverse effects commonly associated with systemic chemotherapy [136, 137].

Despite the promising advantages of LNPs, several limitations must be addressed before the clinical application for OSCC treatment. One of the primary challenges is the complexity of LNPs formulation and characterization. The development of LNPs requires precise control over particle size, surface charge, and lipid composition, as these factors significantly influence the pharmacokinetics, biodistribution, and therapeutic efficacy [138]. Another significant limitation is the potential for immunogenicity and toxicity associated with LNPs. While LNPs are generally considered biocompatible, the introduction of foreign materials into the body can trigger immune responses. Therefore, extensive preclinical studies are necessary to assess the immunoreactivity of LNPs and to optimize their design to minimize adverse immune responses [139, 140]. The scalability of LNPs production is another critical challenge. The manufacturing of LNPs on a large scale requires sophisticated equipment and stringent quality control measures to ensure uniformity and consistency across batches. The high cost and technical demands of LNPs production may limit their accessibility and widespread adoption [141]. Moreover, the stability of LNPs during storage is a concern, as changes in environmental conditions, like temperature and humidity, can influence the integrity [142]. Overcoming these challenges will be crucial for the successful translation of LNPs-based therapies from the laboratory to clinical application. In addition to these technical challenges, the regulatory landscape for LNPs is complex and demanding. The approval process for nanoparticle-based therapies involves rigorous evaluation of their safety, efficacy, and quality, including detailed pharmacokinetic studies, biodistribution analysis, and assessment of potential off-target effects [143]. Given the heterogeneity of OSCC and the variability in patient responses to treatment, personalized LNPs formulations tailored to the specific molecular and genetic profile of an individual’s tumor could offer more targeted and effective therapies. Personalized LNPs therapies could also incorporate patient-specific factors, such as comorbidities and immune status, to optimize dosing regimens and minimize adverse effects [144, 145].

## Conclusion

LNPs represent a transformative advancement in the treatment of OSCC, offering significant potential to overcome the limitations of conventional therapies. The ability of LNPs to enhance drug delivery with site-specific targeting and controlled release opens new avenues for the effective treatment of OSCC. Advances in LNPs design, including surface modification and stimuli-responsive properties, are pivotal in enhancing the accumulation of the drug in the solid tumor, biological half-life, and therapeutic efficacy of these nanocarriers. However, despite the promising preclinical results, the translation of LNPs from bench to bedside faces several challenges, including the need for rigorous clinical trials, scalability of production, and thorough regulatory evaluations. Addressing these challenges requires multidisciplinary collaboration among researchers, clinicians, and regulatory bodies to ensure the safe and effective application of LNPs in clinical settings. Moreover, the incorporation of advanced imaging techniques with LNPs-based therapies can facilitate real-time monitoring and optimization of treatment regimens, thereby improving therapeutic efficacy. Personalized medicine approaches, guided by molecular profiling of tumors, will be crucial in maximizing the benefits of LNPs-based therapies for individual patients. The future of OSCC treatment lies in the successful integration of nanotechnology, personalized medicine, and patient-centered care, paving the way for more effective and less toxic therapeutic options.

## Abbreviations

5-FU: 5-fluorouracil
ATRA: all-trans-retinoic acid
CUR: curcumin
DOX: doxorubicin
DS: droplet size
EE: entrapment efficiency
EGFR: epidermal growth factor receptor
EPR: enhanced permeability and retention
FA: folic acid
HPV: human papillomavirus
LNCs: lipid-core nanocapsules
LNPs: lipid-based nanoparticles
LPHNPs: lipid polymer hybrid nanoparticles
MDR: multi-drug resistance
NLCs: nanostructured lipid carriers
OSCC: oral squamous cell carcinoma
PDI: polydispersity index
PDT: photodynamic therapy
PEG: polyethylene glycol
PE-PEG: phosphatidylethanolamine-polyethylene glycol
PS: particle size
PTT: photothermal therapy
RES: reticuloendothelial system
ROS: reactive oxygen species
SLNs: solid lipid nanoparticles
SNEDDS: self-nano emulsifying drug delivery system
VS: vesicle size
ZP: zeta potential

## Declarations

### Copyright

© The Author(s) 2024.
